# The association between fecal enterotoxigenic *B. fragilis* with colorectal cancer

**DOI:** 10.1186/s12885-019-6115-1

**Published:** 2019-09-05

**Authors:** Fakhri Haghi, Elshan Goli, Bahman Mirzaei, Habib Zeighami

**Affiliations:** Department of Microbiology, School of Medicine, Zanjan University of Medical Sciences, Zanjan, Iran

**Keywords:** *Bacteroides fragilis*, Enterotoxin, *bft* gene, Colorectal cancer, Stool

## Abstract

**Background:**

Enterotoxigenic *Bacteroides fragilis* (ETBF) is an enterotoxin-producing bacterium that possibily has a role in the occurrence and progression of colorectal cancer (CRC) by modulating the mucosal immune response and inducing epithelial cell changes. The aim of this study was to investigate the frequency of ETBF in stool samples of CRC patients and healthy volunteers.

**Methods:**

A total of 60 stool samples from confirmed CRC patients and 60 stool samples from healthy volunteers with no personal or familial history or diagnosis of colorectal disease were collected. Stool samples were screened for direct detection of *B. fragilis* using PCR targeting the marker genes of *neu* and *bft.* Enterotoxin isotypes *bft-1, bft-2* and *bft-3* were also detected in *B. fragilis* positive samples.

**Results:**

The frequency of *B. fragilis* among CRC and control cases was 58.3 and 26.6%, respectively (*P* < 0.05). The rate of *bft* gene in CRC cases was significantly higher than in controls (P < 0.05). Also, the presence of *bf*t gene in CRC patients stage III was significantly higher than stages I and II (*P* < 0.05). Enterotoxin isotype *bft-2* was detected with higher frequency among CRC patients than healthy control (P < 0.05).

**Conclusion:**

Our results show the association between fecal ETBF and CRC, and we suggest that detection of ETBF may be a potential marker for colorectal cancer diagnosis. However, additional investigations on tumor and paired normal tissue samples are required to substantiate this possible correlation.

## Background

Colorectal cancer (CRC) is one of the most common types of cancers, with the fourth highest mortality rate among all cancers worldwide [1, 2]. Diet (high red meat/low fiber), obesity, smoking, alcohol consumption and inheritance are the most important risk factors for initiation and progression of CRC [2, 3]. Recent studies showed the significant association between CRC development and intestinal microbiota [4].The dietary risk of CRC is probably associated with dysbiosis of the gut microbiota and their metabolites [1]. It is supposed that bacterial species such as enterotoxigenic *Bacteroides fragilis* (ETBF), *Fusobacterium nucleatum, Clostridium septicum*, *Enterococcus faecalis*, *Helicobacter pylori*, *Streptococcus bovis* and *Escherichia coli* have a role in colorectal carcinogenesis [4, 5]. It has been shown that secreted bacterial toxins increase the cancer risk via toxin-mediated DNA damage. Furthermore, the release of reactive oxygen species (ROS) and the expression of cytokines and chemokines after bacterial infections can be exacerbate ROS-mediated DNA damage [6, 7].

*B. fragilis* is the most frequent anaerobe isolated from clinical cases of diarrhea, peritonitis, intra-abdominal abscesses and sepsis [1, 3, 8, 9]. Previous studies showed the significant correlation between the presence of ETBF in stool or colonic biopsy specimens and active inflammatory bowel disease and CRC [3, 8–10]. Zinc-dependent metalloprotease toxin called the *B. fragilis* toxin (BFT) cleaves the extracellular domain of cell surface protein E-cadherin and resulting in the complete degradation of the E-cadherin. The cytoplasmic domain of E-cadherin associates with the nuclear signaling protein β-catenin. The loss of E-cadherin triggers ß-catenin nuclear signaling, induces *c-myc* expression and IL-8 secretion [2, 8, 9, 11]. BFT also causes oxidative DNA damage, epithelial barrier damage and activation of STAT3/Th17 immune responses [3, 7]. So, it is possible that long-term colonization of colonic epithelial cells with ETBF may increase the risk of CRC [6]. In apc -deficient mice, BFT induced interleukin 17 (IL-17)-dependent inflammation and distal CRC progression [12, 13]. Previous studies have demonstrated that ETBF level in tumor and stool samples were significantly higher in late stages (III/IV) of CRC compared to control tissues [11, 14]. In study conducted by Toprak et al., the *bft* gene was detected in stool samples of 38% of CRC patients, while it was present in 12% of the samples in control group [8].

The aim of this study was to investigate the frequency of ETBF in stool samples of CRC patients and healthy volunteers to find the possible correlation between fecal ETBF with CRC.

## Methods

### Sample collection

Between March 2016 and February 2018, a total of 60 stool samples were collected from confirmed CRC patients admitted to oncology ward of Valiasr hospital in Zanjan province, Iran. Also, 60 stool samples were collected from healthy volunteers with no personal or familial history or diagnosis of colorectal disease as control group. None of the cases or controls had a previous history of diarrhea and antibiotic therapy within the past 1 month. This study was approved by the Research Ethics Committee of Zanjan University of Medical Sciences (ZUMS.REC.1394.235) and informed consent was obtained from all participants at the time of samples collection.

### DNA extraction

Extraction of DNA from stool samples was performed according to the protocol provided with the GeneAll Exgene™ Stool DNA Mini Kit (GeneAll Biotechnology, Korea). The concentration and purity of DNA samples were determined using a NanoDrop Spectrophotometer (ND-1000, Nano-Drop Technologies, Wilmington, DE) at 260 and 260/280 nm, respectively.

### Detection of *B. fragilis* in stool samples by PCR

Stool samples were screened for direct detection of *B. fragilis* using PCR as described previously [15–17]. The marker genes of *neu* and *bft* (encoding neuraminidase and *B. fragilis* toxin, respectively) were used as species-specific targets. PCR was performed using DreamTaq PCR Master Mix (Ampliqon, Denmark), which contains Taq polymerase, dNTPs, MgCl2 and the appropriate buffer. Each PCR tube contained 25 μl reaction mixture composed of 12.5 μl of the master mix, 1.5 μl of each forward and reverse primer solution (in a final concentration of 200 nM) (Metabion, Germany), 1 μl of DNA with concentration of 200 ng/μl and nuclease-free water to complete the final volume. PCR was performed using the Gene Atlas 322 system (ASTEC, Japan). Amplification involved an initial denaturation at 94 °C, 5 min followed by 35 cycles of denaturation (94 °C, 1 min), annealing (62 °C for *neu* and 52 °C for *bft*, 1 min) and extension (72 °C, 1 min), with a final extension step (72 °C, 7 min). The amplified DNA was separated by submarine gel electrophoresis, stained with ethidium bromide and visualized under UV transillumination (UVITEC, UK). The ETBF strain D-134 was used as the positive control strain.

### Detection of enterotoxin isotype encoding genes of *B. fragilis*

The enterotoxin isotype encoding genes (*bft-1, bft-2* and *bft-3*) were detected in *B. fragilis* positive samples as described previousely [17]. Triplex PCR was performed according to following program: initial denaturation at 94 °C, 5 min followed by 35 cycles of denaturation (94 °C, 1 min), annealing (62 °C, 1 min) and extension (72 °C, 1 min), with a final extension step (72 °C, 5 min).

### Statistical analysis

The data were analyzed with SPSS version 17.0 software (SPSS, Inc., Chicago, IL). Pearson’s chi-square or Fisher’s exact test were used to determine the statistical significance of the data. A *P* value of < 0.05 was considered significant. Relative risk calculation with 95% CI was performed only for 2× 2 tables.

## Results

In our study, a total of 60 stool samples of CRC cases (with a male: female ratio of 30:30) and 60 from healthy control cases (male: female ratio of 30:30) were investigated. The median age of CRC cases was 53 years (range 29–90 years) and for healthy controls was 51 years (range 33–85). The majority of CRC cases were stage II or III cancer (26 (43.3*%*) in stage II; and 23 (38.3*%*) in stage III). Also, 11 (18.3%) CRC cases were stage I. None of the cases or controls had a previous history of diarrhea and antibiotic therapy within the past 1 month.

Direct detection of *B. fragilis* from stool samples was performed based on determination of neuraminidase (*neu*) and *B. fragilis* toxin (*bft*) encoding genes. As shown in Table 1, the frequency of *neu* gene among CRC and control cases was 58.3 and 26.6%, respectively. So, the frequency of *B. fragilis* among CRC patients was significantly higher than control group (*P* < 0.05). Furthermore, the *bft* gene was detected among 19 (31.6%) of CRC cases, compared with 5 (8.3%) of healthy controls. The rate of *bft* gene in CRC cases was significantly higher than in controls (*P* < 0.05). The presence of *bft* gene in stool samples of CRC patients with respect to disease status is shown in Table 2. The presence of *bf*t gene in CRC patients stage III was significantly higher than stages I and II (*P* < 0.05). The frequency of *bft* isotypes (*bft-1, bft-2* and *bft-3*) is shown in Table 3. The frequency of *bft-2* isotype in CRC cases was significantly higher than healthy control group (*P* < 0.05).

**Table 1:** Frequency of *neu* and *bft* genes in CRC cases and control group

Target gene	CRC cases (*n* = 60)	Healthy control cases (*n* = 60)	*P* value	Total number (*n* = 120)
*neu*	35 (58.3%)	16 (26.6%)	0.001	51 (42.5%)
*bft*	19 (31.6%)	5 (8.3%)	0.002	24 (40%)

**Table 2:** Presence of *bft* gene in CRC patients with respect to disease status

Stage of cancer	Stage I	Stage II	Stage III	*P* value
*bft* gene	(*n* = 11)	(*n* = 26)	(*n* = 23)	
*bft* positive (*n* = 19)	2 (18.2%)	5 (19.2%)	12 (52.2%)	0.027
*bft* negative (*n* = 41)	9 (81.8%)	21 (80.8%)	11 (47.8%)

**Table 3:** Frequency of *bft* isotypes in in CRC cases and control group

*bft* isotypes	CRC cases (*n* = 60)	Health control cases (*n* = 60)	*P* value	Total number (*n* = 120)
*bft- 1*	7 (11.6%)	3 (5%)	0.322	10 (8.3%)
*bft- 2*	10 (16.6%)	1 (1.6%)	0.008	11 (9.2%)
*bft- 3*	2 (3.3%)	1 (1.6%)	1.000	3 (2.5%)

## Discussion

Various studies suggest that gut microbial dysbiosis may be related to some disorders such as inflammatory bowel disease (IBD), gastrointestinal cancers, diabetes, obesity, hypertension, renal disorders and etc. [6, 18]. Association between gut microbiota and CRC has been reported in several studies [5–8, 19]. According to previous reports, bacterial species including *Streptococcus* species, *H. pylori*, *E. faecalis*, *B. fragilis*, *C. septicum* and *E. coli* have a role in the occurrence and progression of CRC [2]. *B. fragilis* is the most frequent anaerobe isolated in clinical cases of diarrhea, peritonitis, intra-abdominal abscesses and sepsis [11]. It is also associated with intestinal tumors due to enterotoxin production [2]. It has been proposed that enterotoxigenic *B. fragilis* may act as “keystone” pathogen that facilitate the establishment of dysbiotic microbial communities and induce CRC [6, 20, 21]. In the present study, the frequency of enterotoxigenic *B. fragilis* in stool samples of CRC patients was compared with healthy controls. According to *neu* gene determination, *B. fragilis* was detected more frequently from stool samples of CRC patients than from the matched controls (58.3 and 26.6*%*, respectively; *P* < 0.05). In previous study from Iran, higher numbers of *F. nucleatum, E. feacalis*, *S. bovis*, ETBF and *Porphyromonas* spp. were detected in adenomatous polyp cases, consisting tubular adenoma and especially villous/ tubuvillous polyp, in contrast to samples from the normal groups (*P* < 0.001) [19]. It is reported that over time accumulation of ETBF strains in colonic epithelial crypts may enhance carcinogenesis [14].

In our study, the rate of *bft* gene in CRC cases was significantly higher than in controls (*P* < 0.05). This result supports prior works where *bft* detection in stool and colon mucosal samples were significantly higher in CRC patients than in outpatient controls [8, 14]. According to Boleij et al. study, the mucosa of CRC patients was significantly more often *bft*-positive on left (85.7%) and right (91.7%) tumor compared with left and right control biopsies (53.1%; *P* = .033 and 55.5%; *P* = .04, respectively) [14]. It is assumed that BFT exposure in the human colon may induces rapid onset of chronic IL-17–dependent inflammation, oxidative DNA damage, epithelial barrier damage and activation of STAT3/Th17 immune responses yielding to increased risk of CRC [3, 6, 10, 19].

The presence of *bf*t gene in CRC patients stage III was significantly higher than stages I and II (*P* < 0.05). According to Boleij et al. and Viljoen et al. studies, *bft* was detected in the majority of CRC patients in particular with late-stage disease, possibly due to enhanced anaerobiosis on larger tumors [11, 14].

Furthermore, the frequency of *bft-2* isotype in CRC cases was significantly higher than healthy control group (*P* < 0.05). Similar to our study, Boleij et al. show *bft*-2 as the most common mucosal isotype of *B. fragilis* [14]. According to in vitro and in vivo surveys, BFT-2 has greater potency and biological activity compared to BFT-1 and exhibites enhanced carcinogenic potential [14]. However, in study conducted by Ulger Toprak et al., *bft-1* was detected more than *bft-2* in stool samples of colon cancer patients and control group. The *bft-1* isotype was also found in all isolates of extraintestinal sites in their study [22]. Also, *bft-1* isotype was the most frequent allele among ETBF strains isolated from diarrheal diseases [14, 22].

One of the limitations of the present study was the small size of CRC group. Furthermore, data regarding environmental factors and some clinicopathological and demographic characteristics that may contribute to carcinogenesis were not collected from CRC patients in our study.

## Conclusions

Our study revealed that *bft* gene in stool samples of CRC patients stage III was significantly higher than in controls. Also, the frequency of *bft-2* isotype in CRC cases was significantly higher than controls. Our results show the association between fecal ETBF and CRC, and we suggest that detection of ETBF or *bft-2* isotype may be a potential marker of colorectal cancer. However, additional investigations on tumor and paired normal tissue samples in CRC patients are recommended to substantiate this possible correlation.

## Data Availability

The datasets will not be available on a publically available website, but it may be possible to provide access to anonymized data. Anyone who wants to request the data can contact with Habib Zeighami, corresponding author.

## Abbreviations

BFT: *Bacteroides fragilis* Toxin
CRC: Colorectal Cancer
ETBF: Enterotoxigenic *Bacteroides fragilis*
neu: Neuraminidase
PCR: Polymerase Chain Reaction
